# Changing pattern and etiology of maxillofacial fractures during the civil uprising in Western Libya

**DOI:** 10.4317/medoral.22268

**Published:** 2018-02-25

**Authors:** Mohammed S. Elarabi, Anwar B. Bataineh

**Affiliations:** 1 BDS, MmedSC, FFDRCS, Professor of Oral and Maxillofacial Surgery, Oral and Maxillofacial Surgery Department, Ali Omar Askar Neurosurgery University Hospital, Tripoli, Libya; 2 BDS, MScD, MDSc, CSOS , Professor of Oral and Maxillofacial Surgery, Faculty of Dentistry, Jordan University of Science and Technology, Irbid, Jordan

## Abstract

**Background:**

The purpose of the present study was to evaluate changing pattern in characteristics of maxillofacial fractures and concomitant injuries in Western Libya During revolution and to assess the association between mechanism of injury and fracture patterns.

**Material and Methods:**

A retrospective review of medical records and radiographs of 187 patients treated for maxillofacial fractures from January 2010 to December 2012 was performed, there were 326 fractures in 187 patients.

**Results:**

The male: female ratio was 6:1. Most fractures occurred in patients aged 11 to 40 years, and few injuries occurred in patients aged > 50 years. Most fractures occurred from motor vehicle accidents, and other most frequent causes included assault, gunshot, and fall injuries. Most maxillofacial fractures involved the mandible, zygomatic complex, or maxilla. Most mandibular fractures occurred at the parasymphysis, angle, or condyle. Associated injuries most frequently involved the head, chest, and extremities. Most patients were treated with open reduction (132 patients [71%]), and 26 patients (14%) were treated nonoperatively. There were 21 complications (11%).

**Conclusions:**

In summary, motor vehicle accidents were the most frequent cause of maxillofacial fracture in western Libya, possibly because of the lack of seat belt legislation. Interpersonal violence was a less frequent cause of maxillofacial fracture, possibly because of the religious restriction on alcohol consumption.

** Key words:**Tauma, mandible, zygomatic complex, maxilla, treatment, complications.

## Introduction

Maxillofacial injuries involve soft and hard tissues of the face from the frontal bone to the mandible (1). The maxillofacial region is vulnerable to trauma because it is the most exposed part of the body (2). Maxillofacial fractures may occur alone or in combination with fractures of other bones. Fracture patterns may vary with mechanism of injury, magnitude and direction of impact force, and anatomy of the injured site (3).

Maxillofacial trauma presents as skeletal, dental, and soft tissue (3). The common causes of maxillofacial fractures worldwide are motor vehicle accidents, falls, assaults, firearm injuries, sports, and industrial accidents (4). These causes may vary with geography, socioeconomic status, cultural characteristics, and era (5). Maxillofacial fractures are most frequently caused by motor vehicle accidents in developing countries (6) and interpersonal violence in Western countries (7). The most common causes of maxillofacial fractures in different age groups are motor vehicle accidents in adults and falls in the younger population (8).

Epidemiologic studies have shown that age and sex are important factors that affect the occurrence of maxillofacial trauma (9) The highest incidence is observed in patients aged 21 to 30 years, and the lowest incidence is in patients aged > 60 years and < 5 years5 The male: female ratio worldwide is 4:1 (10).

During the past several decades, major developments have been made in the treatment of maxillofacial fractures, including open reduction and internal fixation.

Maxillofacial trauma is becoming a burden and a leading medical problem in emergency rooms worldwide because of the upward trend in facial injuries associated with changes in population patterns such as increased industrialization and urbanization. Maxillofacial trauma may cause death because of the proximity to the brain and the respiratory and digestive tracts, and concomitant injuries may be fatal. The treatment of fractures of the maxillofacial apparatus remains a challenge to maxillofacial surgeons in developing countries because this usually requires much skill and sophisticated equipment for diagnosis and treatment that frequently are lacking in developing countries.

The purpose of the present study was to evaluate changing pattern in characteristics of maxillofacial fractures and concomitant injuries in Western Libya During revolution and to assess the association between mechanism of injury and fracture patterns.

## Material and Methods

A retrospective review of the medical records and radiographs was performed for 187 patients who were evaluated and treated for maxillofacial fractures at the Oral and Maxillofacial Surgery Department, Ali Omar Askar Neurosurgery University Hospital, Tripoli, Libya. The hospital was a major downtown hospital serving a demographically diverse population of 1.6 million and was a tertiary referral center for all fractures in western Libya. A full assessment of the cause and pattern of maxillofacial fractures in all treated patients was required by government health authority officials for use in prevention programs and education of the population. Between January 2010 and December 2012, we treated 326 facial fractures in 187 patients.

Data about age, sex, cause of fracture, anatomic site, mechanism of injury, associated injuries, treatment, and postoperative complications were reviewed. The cause of injury was classified as motor vehicle accident, assault, gunshot wound, fall, animal-related, sports, iatrogenic, or industrial accident.

After most patients presented in the Oral and Maxillofacial Surgery Department, treatment started with Advanced Trauma Life Support protocols including the maintenance of airway, control of bleeding, antibiotic coverage, regular mouth washes, and liquid diet. In all patients, plain radiographs, orthopantomograms, Water views, or computed tomography scans were obtained when possible. Most patients had surgery during the regular operating room schedule. All patients were operated under general anaesthesia. Most patients were treated with open reduction using titanium miniplates, reconstruction plates, microplates, or biodegradable plates, and the other patients received closed or nonoperative treatment.

Fractures were classified as maxillary, mandibular, and zygomatic complex fractures. When > 1 facial bone fracture occurred in a single patient, the fracture was classified as a combination fracture. Most patients who had isolated nasal bone fractures and dentoalveolar fractures were not included in this study. The fractures were also being classified as fractures of the mandible, zygomatic complex, maxilla, fronto-orbital region, upper dentoalveolar region, nasal bone, isolated zygomatic bone, nasoethmoidal complex, panfacial, and split palate. Descriptive statistics were used to calculate percentages.

## Results

There were 326 fractures in 187 patients who were seen and treated. Most fractures occurred in patients aged (11) to 40 years, and few injuries occurred in patients aged > 50 years, the male:female ratio was 6:1 ( Table 1). Most fractures occurred from motor vehicle accidents, and other most frequent causes included assault, gunshot, and fall injuries ( Table 2). Fractures caused by gunshot mostly were in males during the 2011 revolution.

**Table 1:**
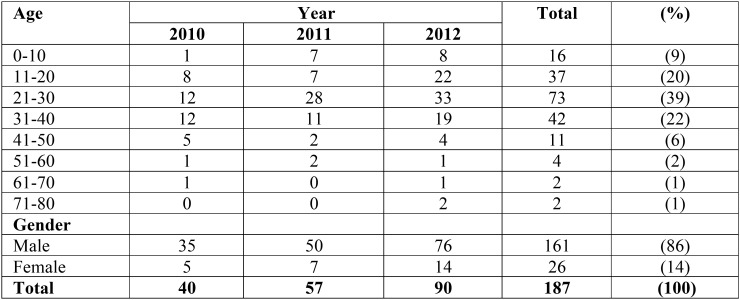
Distribution of fractures according to Age and Gender.

**Table 2:**
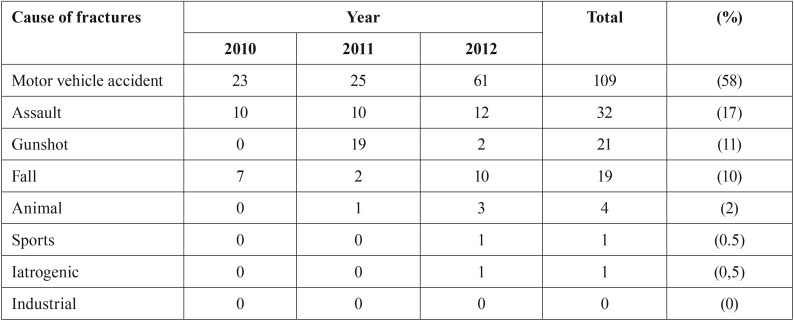
Distribution of fractures according to Causes.

Most maxillofacial fractures involved the mandible, zygomatic complex, or maxilla. Most mandibular fractures occurred at the parasymphysis, angle, or condyle ( Table 3).

**Table 3:**
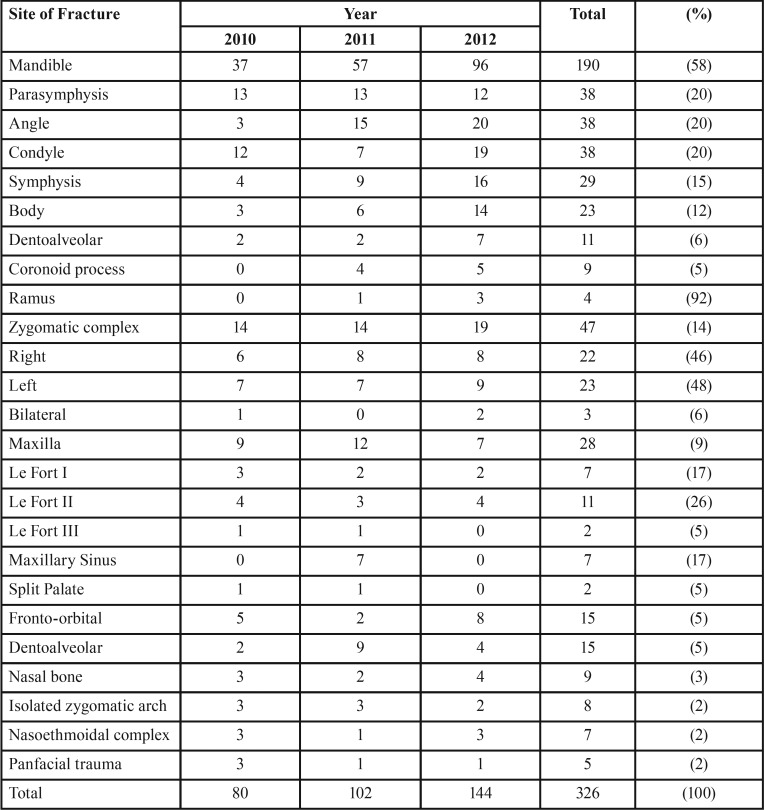
Distribution of fractures according to Site.

Associated injuries most frequently involved the head, chest, and extremities. Treatment in most patients included open reduction and internal fixation with plates such as titanium miniplates, microplates, reconstruction plates, and biodegradable fixation devices. Treatment was refused by 23 patients who discharged themselves against medical advice after diagnosis. Complications (total, 21 patients [11%]) most frequently included nerve symptoms such as numbness, muscle weakness, or paresthesia in 10 patients ( Table 4).

**Table 4:**
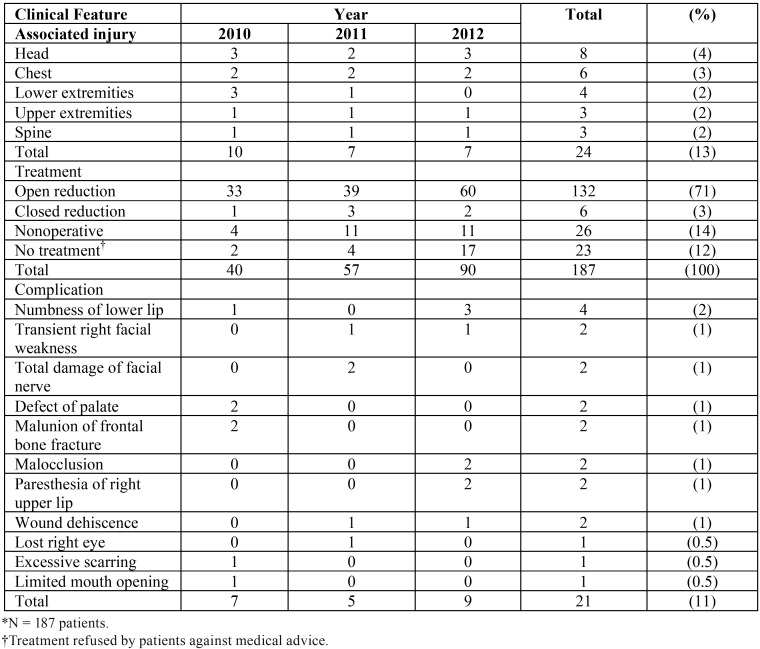
Distribution of fractures according to Clinical Feature*.

## Discussion

The epidemiologic features of maxillofacial fractures are affected by cultural, social, and political factors, with wide variation between or within different countries. Facial bone fractures are a public health issue globally because of associated mortality, morbidity, and major socioeconomic consequences (11-14). The present study provided information that may help guide the planning of preventive programs and maxillofacial trauma care locally and regionally.

Epidemiologic studies have shown that age and sex are important factors that affect the incidence of maxillofacial trauma. The higher male:female ratio in the present study than previous studies may be attributed to the confinement of women to the home and greater exposure of men than women to motor vehicle accidents, fights, industrial work, and sports (12). A high male:female ratio has been reported in other studies worldwide 16 and from the Middle East, (2-17-20) but the ratio is lower in studies from western Europe and Japan (3,12-16,21-23) Recent data showed that the male:female ratio worldwide was 4:1 from 1987 to 2007, the ratio was greater in developing countries (5.1:1) than developed countries (3.7:1). The higher incidence of maxillofacial fractures in males than females may be attributed to higher levels of physical activities in males24 or greater exposure of males to risky behaviors including highway driving, contact sports, and driving after consuming alcohol or drugs (19-24). In addition, in many traditional Arab, African, and Islamic societies, males more often are involved in daily outside work, move to various regions for work, and use highway roads where most accidents occurs. In Libya, women may work outside and drive cars, but they typically limit driving to local areas and not highways or long distances, similar to patterns described in other studies (5,7,9,14,25,27).

Women predominantly take care of the home and typically are not involved in other activities outside the home. In other regions, the women live a more outdoor life and share similar jobs with men and may be exposed to similar risk factors (12,16-21).

The highest incidence of maxillofacial fractures observed in patients aged 21 to 30 years is consistent with previous studies (11-16,24,29).

This age group may include people who have marked physical energy and adventurous behavior but lack experience associated with older age, and they may be more likely to disregard traffic regulations than older people (11,13-25). In the present study, the youngest patient was aged 18 months and the oldest patient was aged 78 years. Patients aged 21 to 30 years have completed postsecondary education and typically make numerous road trips in search of employment. The increasing number of motor vehicle accidents in developing countries such as India may be attributed to many factors such as sharing of roadways between pedestrians, animals, and fast- and slow-moving vehicles, with limited segregation of pedestrians from wheeled traffic; the large numbers of old and poorly maintained vehicles on roads; large numbers of motorcycles, scooters, and mopeds; low driving standards; large numbers of overloaded buses; widespread disregard for traffic rules; defective roads; poor street lighting; and defective layout of crossroads and speed breakers (3). In addition, the increased traffic volume because of economic expansion, and the rapid increase in the density of urban populations, may add to the incidence of motor vehicle accidents. Improved education about road safety may decrease the incidence of motor vehicle accidents (12,14,16,25-28).

In several studies, motor vehicle accidents were the major cause of maxillofacial injuries (12,14,17,18,28). The present results were consistent with findings of other studies (16,18,22-29). Vehicles that have 2 wheels, such as bicycles and motorcycles, are less stable than cars and provide little protection to drivers in accidents. This may be the possible explanation for the increased frequency of motor vehicle accidents involving 2-wheeled vehicles. The increasing cost of vehicle spare parts may cause vehicle owners to seek substandard alternatives, and this may compromise the safety of the vehicle and driver or the passengers in collisions with heavy vehicles. Most commercial vehicle drivers are illiterate and may not be able to read and properly interpret simple road signs. This may be the reason for higher involvement of buses and trucks than cars in motor vehicle accidents in previous studies (11,16,27).

The increasing frequency of fights and assaults may be attributed to increasing interpersonal violence associated with alcohol consumption and unemployment. Studies vary in the frequency of maxillofacial injuries attributed to falls, daily activities, sports, fights, and assaults (19,22,23). The causes of maxillofacial injuries may vary between countries. Fights, assaults, falls, and animal injuries were less frequently the cause of facial injuries in the present than other studies.

In the present study, gunshot wounds became the second most frequent cause of facial injuries in 2011. In February 2011, a revolution started in Libya against the old regime, and there was armed conflict between the army and rebels. There were no official figures available of gunshot-related injuries or deaths during the revolution. The present study suggests that maxillofacial injury from gunshots has very low frequency, similar to Western countries.

The more frequent involvement of the mandible than other maxillofacial sites may be attributed to the anatomic prominence and exposed anatomic position of the mandible on the face. During a motor vehicle accident, most victims may try to avoid injury to the head and may receive maximum impact to the mandible, causing higher risk of fracture to the mandible than other facial bones. The enforcement of strict laws to make seat belts mandatory would reduce the incidence of maxillofacial trauma by decreasing trauma from the dashboard, steering wheel, or windshield. In a retrospective study of maxillofacial fractures in 563 patients during 5 years in Jordan, the mandible was most frequently fractured (74.4%) followed by the maxilla, zygomatic arch, and dentoalveolar process (2). The most common causes of injury were motor vehicle accidents (55.2%), accidental falls (19.7%), and assault (16.9%). Other studies also showed that mandibular fracture was the most common maxillofacial injury (11,12,24,27,30). The force of a blow is transferred from the chin along the mandible to the condyle and may cause fractures in the neck of the condyle, which is a weak anatomic site within the mandible. The parasymphysis and angle also are weak anatomic sites that are susceptible to fracture because of the long roots of canines, presence of third molars, and abrupt change in the direction between the large, strong body of the mandible and the thin ascending ramus (6,11,17,20,24,29). In the present study, the mandible was most frequently fractured at the parasymphysis, angle, and condyle, consistent with the results of previous studies (2,11,12,24,30).

The most frequent midfacial fractures, in decreasing order of frequency, include zygomatic complex, Le Fort, and dentoalveolar fractures. In the present study, zygomatic complex fractures were the most common midface fractures, followed by nasoethmoidwed by panfacial, dentoalveolar, and Le Fort fractures. Isolated blowout and nasoethmoid fractures accounted for < 7% fractures. Other studies showed that zygomatic complex fractures are the most frequent midfacial fractures, but there is variation in the frequency of fractures of the other midfacial bones (26-28). Zygomatic complex fractures may occur because of the instinctive turn of the head when anticipating a blow to the midface to protect the eye. A previous study showed that the zygomatic complex was the most common site of middle face injury (23).

Alcohol consumption by youths is increasing, and alcohol impairs driving ability and increases the risk of an accident. Drugs such as barbiturates, amphetamines, and cannabis impair the ability to drive safely. Alcoholism is associated with violence, including fights and assaults that cause maxillofacial injuries in male alcoholics, as previously reported (13,16,19,21).

Government authorities should consider banning alcohol and other drugs (12). The weekend parties and excessive use of alcohol among youths may be responsible for the high incidence of accident-related maxillofacial injuries on Saturdays. Therefore, there is a need to emphasize the importance of common restraint devices and good road habits, especially during this high-risk period, to reduce the incidence of maxillofacial fractures caused by motor vehicle accidents. The present results are in accordance with previous studies (13,16,19,21).

Previous studies showed that the frequency of maxillofacial injuries was highest in either January or June to August (19,20). The timing of accidents in nonalcoholics coincided with peak traffic hours and the time when most alcoholics return home.

Skull, chest, neck, and spine injuries frequently are associated with maxillofacial fractures in severely injured patients. Knowledge about these associated injuries may provide useful strategies for patient care and prevention of further complications. A multidisciplinary and coordinated approach may provide optimum stabilization and treatment of patients who have facial fractures. Associated injuries usually are severe and serious and reflect the severity of injuries caused by high-speed motor vehicle accidents or use of weapons. In the pre-sent study, facial fractures occurred in combination with other injuries to the head, chest, lower and upper extremities, and spine, as previously reported. Therefore, immediate diagnosis and coordination of care are important from general, orthopedic, plastic, maxillofacial, neurological, ophthalmic, and dental surgical teams. Most patients in the present study had associated injuries treated concomitantly. These features were not included in previous studies from Pakistan (13,16,24,29). The relation between maxillofacial and other injuries is evidence that it is necessary for the maxillofacial surgeon to be part of a multidisciplinary trauma team. This may minimize delays in consultation and referral and ensure that maxillofacial injuries are treated promptly and concurrent with associated injuries.

Treatment options may depend on the type and extent of the fracture and other concurrent problems. During the past several decades, there have been major advances in maxillofacial fracture care, including a change from closed to open reduction and internal fixation of facial fractures. The treatment of panfacial fractures has undergone several changes in the past decade, including improvements in plate and screw fixation.

Most patients in the present study were treated with open reduction and fixation with plates and other alloplastic materials (132 patients [71%]), and few patients had closed reduction with arch bar fixation (6 patients [3%]), consistent with a previous study (23,27).

In other studies, closed reduction with arch bar fixation was the main treatment, and only few patients were treated with open reduction and plate fixation (17,28,30). There is controversy about the treatment of fractures, with studies recommending closed reduction or open reduction with rigid internal fixation. In the present study, some patients refused treatment despite diagnosis and recommendations for treatment. Treatment methods may include nonoperative treatment, Gilles temporal approach, the intraoral approach, and direct internal fixation using titanium miniplates (12-15).

In other studies, from Jordan and Nigeria, the major treatment included antral packing, transosseous wiring, and other techniques based on affordability, availability, simplicity, safety, and skill of the surgeon (5,12). In regions with low socioeconomic status of patients, limiting factors may include the high cost of obtaining the necessary specialized training and skill and limited operating room space for treatment under general anesthesia.

In the present study, the frequency of postoperative complications (11%) was lower than that reported previously (18% to 62%) (24). Skull fracture is the most common cause of facial nerve injury, which may occur immediately after injury or several days later because of nerve swelling. Injury to the facial nerve may occur during operations on the ear. The present study showed that transient facial weakness and total damage of the facial nerve occurred in several patients with associated skull trauma. Malunion may occur because of inadequate occlusal or osseous reduction during surgery, absence of osseous reduction, imprecise application of internal fixation devices, or inadequate stability.

In summary, motor vehicle accidents were the most frequent cause of maxillofacial fracture in western Libya, possibly because of the lack of seat belt legislation. Interpersonal violence was a less frequent cause of maxillofacial fracture, possibly because of the religious restriction on alcohol consumption. National collection of data may be useful for planning prevention, legislation, and resource allocation for the treatment of facial fractures. Complications of maxillofacial fractures were associated with fracture complexity.
